# The Use of Negative Acceleration as Accessory Force during Lifting

**DOI:** 10.1155/2018/9164590

**Published:** 2018-12-02

**Authors:** Jordan Trafimow, Alexander S. Aruin

**Affiliations:** Department of Physical Therapy, University of Illinois at Chicago, Chicago, IL, USA

## Abstract

**Objectives:**

Injury associated with lifting, especially low back injury, is a big problem in industry that accounts for loss of work and high medical expenses. Studies of biomechanics of lifting provide a basis for optimization of lifting. The aim of the study was to further investigate the role of the upward force due to negative acceleration during a lift.

**Methods:**

Nine healthy subjects lifted an empty box and a box with additional load of 10, 20, and 25 lb. Kinematic data were recorded during the lifts and accelerations were calculated, and angular positions of the trunk and knee were obtained during the lifting when negative accelerations were used.

**Results:**

Negative acceleration assisted the quadriceps when the thighs were at approximately 90° and the hips when the trunk was rotating toward standing position. Negative acceleration was present during lifts of different loads.

**Conclusion:**

The outcome of the study suggests that enhancing the use of negative acceleration could be a strategy to improve the quality of lifting and minimize a probability of low back injury.

## 1. Introduction

Lifting an object is a routine handling activity in many warehouse and distribution settings, manufacturing environments, in the service and delivery industry, and in patient-handling activities as well as during leisure times. Injuries associated with such activities are widely acknowledged as being an important problem [1]. Indeed, lifting-related injuries, especially low back injury that frequently occur in industry, are associated with low back pain and discomfort and account for most of the time loss and because of that are costly [2]. Postinjury low back pain is a leading problem world-wide [3]. Moreover, it is a leading cause of lost workplace productivity, absence from work, and reduced quality of life [4]. It was also reported that recurrences of low back pain at 1 year after injury range from 24% to 80% [5]. The total healthcare cost associated with treatment of low back pain in the United States exceeds $100 billion per year [6].

Prevention of injuries associated with lifting is recognized as an important aim to maintain occupational health [7]. However, despite the well-documented role of lifting in low back injuries, the literature on safer lifting techniques remains controversial [8]. Nevertheless, it is known that squat lift (when back straight and knee bent) is safer than the stoop lift (when back bent and knee straight) as the load is closer to the body and, therefore, the extra demand on back muscles to counterbalance additional moments is reduced. Moreover, literature suggests that the risk of injury may be influenced more by the lumbar posture rather than the choice of stoop or squat technique [9, 10].

Lifting an object is a complex task requiring consideration of many issues to avoid injury or minimize its probability [11]. Thus, a number of risk factors were identified that are associated with the increased probability of injury as a result of performing of manual materials handling tasks. Among them are weight lifted, frequency of lift, lifting techniques, and trunk twists [12]. Trunk motion and velocity of trunk movement have also been recognized to be significant risk factors in the occurrence of occupational low back disorders [13, 14]. Moreover, stoop, squat, and semistoop techniques have been investigated thoroughly [7]. The outcome of a number of studies provided important information that could be used in the optimization of lifting maneuver. For example, it was described in the literature that using back belt was associated with a reduction of low back injury incidents [15–17] and that lifting a box while rotating it with one edge staying on the ground could lessen the force used for the lift [18]. Moreover, it was reported that work-related measures, such as the total net muscle work, total absolute net muscle work and work done to the load, diminished considerably with the increase of the lifting speed [19]. In addition, the ability of lifters to maintain their balance during a lift should be considered as well [11]. Furthermore, biomechanical models, physiological models, psychophysically based capacity models, safe load handling models, and simulation models were developed to optimize the performance of the activities involving manual materials handling and lifts [20]. Baechle and Earle [21] call attention to situation when a standard weight-lifting technique increasing upward momentum by a sudden maximum effort [21]. This maximum effort may explain the build-up of upward maximum velocity when the lifter lifts the load off the ground, regardless of the weight to be lifted.

It was reported that lifters use negative acceleration to assist in lifting while maintaining upward (positive) velocity [22]. However, the conclusion was made based on lifting only very light weights. The objective of the current study was to further investigate the role of negative (downward) acceleration in assisting lifting of larger weights. Downward acceleration is equivalent to upward force [22]. This upward force is called the “D force” after D'Alembert [23], who provided the first step in the derivation (see Appendix).

Our hypothesis was that lifters use D force to assist the motion of the knees and the trunk during lifting of objects of different weight. Specifically we hypothesized that D force will be generated during lifting of 0 lb., 10 lb., 20 lb., and 25 lb. objects.

## 2. Methods

### 2.1. Subjects

Nine healthy young adults with no previous experience with weight lifting (5 males, 4 females, 27.4±5.0 years old, height 161.7 ±8.4 cm, and body mass 64.4±15.1 kg) participated in the study. All subjects were right-hand dominant. All subjects provided a written informed consent approved by the Institutional Review Board of the University of Illinois at Chicago.

### 2.2. Procedure

The participants stood with their feet shoulder width apart and lifted a plastic box (weight 0.5 lb, 32 cm long, 50 cm width and 13 cm height) with weights of 0 lb., 10 lb., 20 lb., and 25 lb. added to the box. For each lift the subjects bent down and grasped the box on the floor. The subject then straightened up while bringing the box to elbow height. After they returned to standing, they were prompted to return the box to the ground. Each participant lifted the box one time at each weight level, free style, and in random order.

### 2.3. Instrumentation

A six-camera VICON Motion Analysis 612 system (Oxford Metrics, UK) was used to collect three-dimensional kinematic data at a sampling frequency of 100 Hz. Retroreflective markers were positioned on various anatomic landmarks based on the Plug-In-Gait (PIG) model (Oxford Metrics, UK). The markers were positioned over the lateral border of the arm (between the humeral epicondyle and the acromioclavicular joint markers), acromioclavicular joint, second metacarpal, lateral epicondyle of the humerus, the lateral border of the thigh (between the femoral epicondyle and anterior superior iliac spines), anterior/posterior superior iliac spines, the lateral border of the leg (between the lateral malleolus and femoral epicondyle markers), second metatarsal head, calcaneus, lateral malleolus, lateral epicondyle of the femur. In addition, markers were attached over xiphoid process of the sternum, between the 2 sternoclavicular joints, inferior angle of the right scapula, 10th thoracic vertebra, and 7th cervical vertebra. Moreover, two markers were attached to the wrist bands and four markers were attached to the head band.

### 2.4. Analysis

The initial processing the kinematic data was performed with Vicon and BodyBuilder 3D modeling software. The Plug-In-Gait (PIG) model consisted of fifteen rigid body segments, including feet (2), tibia (2), pelvis, femur (2), hands (2), radius (2), humerus (2), thorax, and head. Height and body mass and 7 anthropometrical measures such as hand thickness, shoulder offset, wrist, elbow, ankle, and knee width as well as leg length for each participant were inserted in the PIG model.

The kinematic data together with these measures were used to calculate the center of mass (COM) displacement and changes in the angular position in major joints. The trunk movement was characterized as the movement of the C7 marker in the rigid body model. Location of the hip joint was obtained using the Newington-Gage model thigh angle [24].

The kinematic data were filtered with a low pass 4th order Butterworth filter with a cutoff frequency of 2 Hz. The COM acceleration was obtained by differentiating the COM position, filtering the resulting velocity trajectory with a low pass 5th order Butterworth filter with a 10 Hz cutoff frequency and then differentiating the COM velocity once and filtering it with a 5th order low pass Butterworth filter with a 10 Hz cutoff frequency.

For analysis purposes, the body was divided into 3 segments. Each segment was considered to be a single rigid body. Segment 1 was the entire body above the horizontal plane between centers of the femoral heads. Segment 2 was between segment 1 and the tibial plateaus and segment 3 was below the tibial plateaus. The trunk angle was calculated as the angle between segment 1 and segment 2 in relation to horizontal axis (0 deg, theoretical angle when segment 1 and segment 2 are parallel). The knee angle was calculated as the angle between segment 2 and segment 3 (0 degree corresponded to the horizontal position of the segment 2 and vertical position of the segment 3).

We recorded the angles at the hips (between the thigh and trunk) and knees (between the thigh and shank) at each time point at which “D” force was present. Time points were recorded as follows: Recordings began when the lifter started the lift, but the data were useful only when D force first appeared. The last record was the last data point at which D force was present. We divided the time between these two points into 100 equally long time intervals, separated by time points. A repeated measure ANOVA was used to compare angles when “D” force was observed (p < 0.05).

## 3. Results

All the subjects used “D” force when lifting a box with 0 lb., and when 10 lb., 20 lb., or 25 lb. loads were added. “D” force was generated over a relative substantial part of the lift.

When lifting objects of different weight, the “D” force was first generated when the angle in the trunk was 39.44±12.85 deg; the “D” force was observed till the trunk angle 94.30±3.52 deg (Table 1). During lifting of different loads (0, 10, 20, and 25 lb), difference between the initial angles in the trunk when “D” force was generated was not significant (p=0.81); the difference between the final angles of the trunk when the “D” force was seen was also not significant (p=0.10).

The angle in the knee when the “D” force was generated first varied from 74.41±13.2 deg to 80.81±11.68 deg. The “D” force was observed till the knee angle 96.66±3.45 deg (Table 1). The difference between these initial angles in the knee measured during lifting of different loads was not significant (p=0.72). Similarly the difference between the final angles in the knee when the “D” force was observed was not significant (p=0.19).

## 4. Discussion

The results of the current study demonstrated that subjects used “D” force when lifting an empty box as well as the box with 10 lb., 20 lb., and 25 lb. load added. Overall, the “D” force was generated when the trunk angle reached 44 degrees (average angle across different loads).

The “D” force was not generated after the trunk angle reached 90 deg. At the knee, the “D” force was first seen at about 79 degrees (across different loads) so upward velocity must be generated before this angle. The “D” force was not generated after the knee angle reached 95 deg: the moment arm is shortening at this point, so “D” force at the knee is not needed further (see Table 1).

It was reported in the literature that D force could be utilized during performance of a “Clean and Jerk” lift (lift of the load from the ground level to overhead position). During this maneuver the lifter applies a sudden maximum effort to create more upward force [21]. This example illustrates the existence of the D force utilized by lifters during a maneuver, different from the task we studied (lifting the load from the floor to waist level). As such, the results of the current study taken together with prior publications allow concluding that the use of D force could be productive while performing different types of lifts.

The observed substantial range in the angular position of the trunk and relatively smaller range in the angular position of the knee suggests that while subjects generated D force during the knee extension, the largest portion of D force was generated during the trunk extension. Indeed, it looks like subjects lifted objects of different weight using a similar strategy generating D force to extend the knee across the 90° (horizontal plane), where the moment to gravity is largest. The data show that D force was seen during the trunk rotation for a much larger angular range than during the knee extension.

The outcome of a prior study [22] revealed that people use D force when lifting very light objects. The results of the current study demonstrated that lifters use D force while lifting objects of different weights. Thus, the results of two studies taken together provide support to the fact that lifters use negative acceleration to build up as much upward trunk velocity as possible before the load clears the ground in order to increase the amount of available D force. It is possible that such a strategy is used to improve the quality of lifting. Moreover, using additional D force might create conditions when the task of lifting an object is biomechanically more efficient and safer. Furthermore, the utilization of D force generated by the quadriceps could be beneficial to individuals with back weakness and/or pain. In addition, the outcome of the study suggests that enhancing the use of negative acceleration could be a strategy to improve the quality of lifting that potentially can prevent low back injury.

While the outcome of the current study confirmed that lifters use D force while lifting objects of different weights, the maximal weight of the object was 25 lb; thus additional studies are needed to confirm that D force is utilized wile lifting heavier objects.

## Figures and Tables

**Table 1:** Trunk and knee angles when “D” force is generated.

Load	Trunk first	Trunk last	Knee first	Knee last
0 lb	49.90±13.99	92.01±9.25	74.41±13.2	96.66±3.45
10 lb	42.80±9.39	94.3±3.52	80.81±11.68	94.79±3.11
20 lb	44.87±7.23	92.23±3.87	80.81±11.68	94.79±3.11
25 lb	39.44±12.85	83.2±15.29	79.43±12.52	91.81±6.08

## Appendix

This Appendix consists of three parts (Appendix has been previously published in [22]).

The first part is an introduction of the approximation of acceleration at each data point x.

CM (x) = center of mass at time point x, similarly for CM(x+1) and CM(x-1).

By definition a(x) = d^2^x/dt^2^, which we approximate by

  [CM(x +1) – CM(x)] – [CM(x) – CM(x-1)] / dt^2^ = a(x).

We presume that [CM(x +1) – CM(x) = d(x) between x and x +1.

Likewise, we presume that

  [CM(x) – CM(x - 1)] = d(x) between x and (x-1).

Therefore, [CM(x +1) – CM(x)] – [CM(x) – CM (x-1)] / dt^2^ = a(x)

if [CM(x +1) – CM(x)] > [CM (x) – CM (x-1)] then a(x) is positive

if [CM(x +1) – CM(x)] < [CM (x) – CM (x-1)] then a(x) is negative.

The second part is the mathematical explanation showing that downward acceleration decreases the net downward force of the weight being lifted. From Newton's law, F = ma (force = mass x acceleration). By D'Alembert's Principle [23] F – m a = 0. This means that (-ma) acts like a force, since it is subtracted from a force. Since a is downward acceleration, (-ma) is upward force. In this study we call (-ma) the D'Alembert force, or D force.

Third, velocity does not appear in Newton's law. If velocity is large enough, the center of mass will continue moving upward, while the negative acceleration decreases the effective force of gravity. An example of positive velocity continuing when there is negative acceleration is an automobile remain moving forward when the driver applies the brake.
